# Impact of the WHO FCTC on non-cigarette tobacco products

**DOI:** 10.1136/tobaccocontrol-2018-054346

**Published:** 2018-07-31

**Authors:** Ghazi S Zaatari, Asma Bazzi

**Affiliations:** Pathology & Laboratory Medicine, American University of Beirut, Beirut, Lebanon

**Keywords:** FCTC, impact assessment, waterpipe tobacco, smokeless tobacco, non-cigarette tobacco products

## Abstract

**Introduction:**

This paper investigates to what extent Framework Convention on Tobacco Control (FCTC) parties have successfully implemented regulatory measures against non-cigarette tobacco product (NCTP) use, considers the challenges and peculiarities in applying such regulations and proposes effective means.

**Data and methods:**

This review was based on many sources mainly: International Legal Consortium, International Tobacco Control, Campaign for Tobacco-Free Kids, FCTC, expert group visits and published literature.

**Findings and conclusion:**

The FCTC provided a framework that applies to all forms of tobacco and this encouraged some parties to adopt control measures against NCTP and to incorporate them into their national tobacco control plans. Although a number of countries have adopted measures specifically targeted towards smokeless and waterpipe tobacco, greater global progress is needed. The strongest achievements have been in protection from exposure to tobacco smoke; controlling advertising, promotion and sponsorship; controlling sales to and by minors; education, communication and public awareness; and packaging and labelling of NCTP. Countries which adopted broad definitions of tobacco products have demonstrated encouraging trends in curbing their use. Future work should address the deep-rooted social acceptance of NCTP, the laxity in their control, their exclusion from regulations in some countries and the failure to subject them to increased taxation. Control measures should also specifically target the initiation risk to youth and adolescents and all factors that contribute to that such as banning flavourings and promotions through social media. Stronger global surveillance of NCTP use, tracking of policy implementation and evaluation of policy impact will provide important evidence to assist parties in fully implementing the FCTC to control their use.

## Introduction

WHO Framework Convention on Tobacco Control (FCTC) is a landmark global treaty to combat the use of all tobacco products. Its articles have provided parties with valuable evidence-based guidance to strengthen the implementation of effective tobacco control measures.^1–3^ Being the most widely used tobacco product, the emphasis of regulations has understandably focused on cigarettes. National tobacco control policies on other forms of tobacco use have lagged behind those on cigarettes. The use of other forms of tobacco, such as waterpipe (WP) and smokeless tobacco (SLT), has grown dramatically in countries that traditionally have high usage prevalence rates, and has extended to countries and regions where their use has been limited or unknown.^4 5^ This pattern of use has been observed because of deep-rooted social and cultural acceptance of some of these products, the perception of less or no harm, affordability and low taxation rates, and the marketing by the tobacco industry of some as short-term substitutes for smoking to evade policies on smoke-free environments or useful cessation tools.^4 5^ This paper reviews the extent to which parties to WHO FCTC have successfully implemented regulatory measures to control the use of non-cigarette tobacco products (NCTPs), identifies the challenges and peculiarities in applying such regulations to the use of selected products, and proposes effective means of regulating them for consideration by FCTC parties.

## Methodology

This paper is a narrative synthesis of global data and published work on the control of NCTP from the following sources: (1) all parties’ submissions to WHO FCTC Secretariat on progress of implementation on tobacco control in the 2016 reporting cycle as per article 21 of the Convention^3^; (2) the global evidence review conducted by the International Tobacco Control Project and data of the International Legal Consortium of the Campaign for Tobacco-Free Kids^6–8^; (3) transcripts and summaries obtained from the 12 country missions undertaken by WHO FCTC Impact Assessment Expert Group^9^; (4) global WHO and FCTC advisories and reports on WP, SLT tobacco and bidi use and (5) published literature between 2002 and 2018 in English language using data bases of National Center for Biotechnology Information, PubMed, MEDLINE, Cochrane, Embase, ProQuest, Global Health and Google Scholar. Keyword searches included: WP, hubble bubble, shisha, hooka, goza, arghile and narghile; as for SLT: chewing tobacco, dip, oral tobacco, snuff, spit tobacco, snus, NCTP, gutka, chewable as well as keywords such as FCTC, systematic review, impact assessment, policy, law, legislation and interventions. 

A narrative synthesis was deemed most appropriate for the purpose of this brief review. The paper describes the extent of implementation of FCTC articles to NCTP globally and by WHO geographical regions, summarises the NCTP regulations data of the 12 country missions undertaken by WHO FCTC Impact Assessment Expert Group, and discusses the published literature on this subject. The database search was limited to locating studies on NCTP and control measures. Evidence from more recent published reviews and systematic reviews was prioritised. Substantiated evidence from multiple sources to account for inherent biases in the methodologies of the data sources was sought. One author (AB) screened and sorted the extracted studies. After removing duplicates, the database search retrieved 412 studies that were screened by title and then by abstract. Of the 412 screened studies, 148 addressed SLT, 135 discussed FCTC in general, 103 covered WP smoking and the remaining 26 studies addressed e-cigarettes and other unrelated topics and tobacco products.

The paper analyses to what extent the FCTC has been instrumental in making a difference in tobacco control efforts. The discussion will highlight the achievements made by parties in their control of NCTP, the peculiarities of these products that require special regulatory measures and the challenges that are to be overcome for effective implementation of FCTC.

One author (GSZ) drafted the manuscript. Both authors revised the manuscript. Writing of the manuscript also built on GSZ’s knowledge from being a member of WHO FCTC Impact Assessment Expert Group.^9^

### Global status of NCTP policy implementation

Although the FCTC pertains to all tobacco products, including NCTPs, global regulations tend to primarily focus on cigarettes. A 2015 WP advisory note by WHO Study Group on Tobacco Product Regulation (TobReg)^5^ found that WP venues and products in low-income and middle-income countries are exempt from tobacco control policies and are poorly enforced where they do exist. TobReg recommended article-specific policies for controlling the use of WP tobacco products. However, regulatory peculiarities unique to this type of tobacco product, such as the varying shapes and sizes of the WP apparatus, and social and cultural factors continue to impede implementation of WP-specific policies and regulations.

In the 2016 cycle, the reporting system on implementation of the FCTC included, for the first time, specific questions for FCTC parties on policies related to new and emerging tobacco or nicotine products.^10^ Data reported in the 2016 Global Progress Report on implementation of FCTC indicated that remarkably few parties have adopted and implemented comprehensive policies or regulations specific to SLT or WP tobacco. Among reporting parties with SLT available on the market, 64% reported that they had SLT policies or regulations in place. Among reporting parties with WP tobacco available on the market, only 57% reported that they had WP-specific policies or regulations.

A recent progress report by the Knowledge Hub on SLT offers a detailed status of the implementation of FCTC articles to SLT products and confirms the findings of the 2016 Global Progress Report as to the progress achieved but yet the low compliance rate in fully implementing them.^11 12^ Of the 179 Parties surveyed, a progressive increase in number of parties applying warning labels to SLT products from 6 (2%) in 2005 to 91 (51%) in 2016 is reported; 27% have large health warnings (at least covering 50% of the SLT tobacco package) and 20% had pictorial health warnings. Banning advertisement is observed in 60% of the surveyed parties with variable and lower rates for banning promotion and sponsorship. Only 8% (15 parties) had a comprehensive ban on SLT tobacco advertising, promotion and sponsorship. Sales to minors is banned in 120 parties (67%) but with lower and variable implementation of all provisions of article 16. As for taxation, the report reaffirms the limited knowledge on the nature of taxes on SLT products or the extent to which higher SLT taxes translate into higher SLT prices, and how these prices affect the consumption and affordability of SLT products, particularly in light of the heterogeneity of these products and the restricted availability of data to the traditional sales markets which is not inclusive of the cottage industry market. Of 32 parties surveyed on taxation, 19 had unit price of SLT products at least two international dollars at purchasing power parity lower than that of cigarettes, thus pointing out the affordability of SLT products in countries with high prevalence of use. Only four parties had tax incidence of 70% and above, and seven parties had no tax of any kind. Implementation of regulations on manufacture, import and sale of SLT products is limited with banning rates of 6%, 3% and 25%, respectively. Only four parties had total ban on manufacture, import and sale of SLT. The report concludes by suggesting appropriate and specific regulatory measures to enhance their implementation and urges the establishment and maintenance of an updated database of laws and regulations on SLT control to be shared among parties for stronger and cooperative regional and global control programmes.^12^

## Regulatory experience in the 12 FCTC impact assessment countries

In 2015, a seven-member WHO FCTC impact assessment expert group (EG) was formed by the Bureau of the Conference of the Parties (COP) to conduct an assessment of the impact of WHO FCTC after its first 10 years of operation as called for at the sixth meeting of the COP (decision FCTC/COP6(13)).^13^ Between November 2015 and May 2016, the EG conducted country missions to two FCTC countries in each of the six WHO regions to assess the relationship between the treaty and policy action in each of the FCTC policy domains.^9^ The impact assessment report by the EG noted increasing recognition among parties of the need to regulate the growing prevalence of SLT and WP tobacco use in several countries leading to some stronger regulations (see table 1 for overview of key policies) such as introducing bans on the importation and/or sale of products (eg, Sri Lanka, Iran) introducing laws requiring large pictorial/text warnings (eg, Turkey, Kenya, Bangladesh, Philippines), banning WP use in public places (eg, Pakistan, Turkey), restrictions on advertising, promotion and sponsorship (Madagascar), and introducing strong price and tax policies (Bangladesh). However, stakeholders expressed ongoing challenges with enforcement and compliance. The EG’s report identified the need for further action, particularly to reduce the affordability of these products.^9^ The section below provides further examples beyond the 12 mission countries, thus offering a broader scope of implementation of NCTP policies across WHO Regions.

**Table 1:** Smokeless tobacco (SLT) and waterpipe (WP) policies in 12 countries selected for FCTC impact assessment

Country (WHO region)	FCTC ratification	SLT	WP
Kenya (AFR)	2004	No SLT-specific regulation; however, national tobacco control laws apply to all tobacco products. Pictorial health warnings required on 30% front/50% back. Comprehensive ban on tobacco advertising, promotion and sponsorship. Complete provisions on banning sales to minors, including all provisions under FCTC article 16.^1^	Ban on import, manufacture, sale, use, advertising, promotion and distribution of shisha (under legal challenge as of June 2018).
Madagascar (AFR)	2004	Pictorial warnings on 50% (front)/50% back of package (snuff). Text warning on 50% (back) of package (chewing tobacco). Comprehensive ban on advertising, promotion and sponsorship. Impose ad valorem excise and value added tax/sales tax on chewing tobacco. Ban on sales to minors. ­ ­ ­ ­	WP tobacco is not available in the national market.
Brazil (AMR)	2005	Legal sale is only permitted if retailer is registered with Brazilian Health Regulatory Agency. No retailers are registered as of June 2018. Any products registered for sale would be subject to provisions of national tobacco control laws, including mandated health warnings.	Ban on use of WP in enclosed common areas. Advertising of WP banned except display at place of sale (text/pictorial warning required on 30% of one surface of display stand/sample case). Text warning required on 30% front and pictorial health warnings required on 100% back of WP tobacco package.
Uruguay (AMR)	2004	No SLT-specific regulation; however, national tobacco laws apply to all tobacco products, including mandated pictorial health warnings on 80% front/80% back. Ban on sales to minors.	No WP-specific regulation; however, national tobacco laws apply to all tobacco products.
Pakistan (EMR)	2004	Use is prohibited at places of public work or use, and in public transport vehicles. Ban on print, electronic and outdoor media advertising of tobacco and tobacco products. Ban on free goods, cash rebates, free samples discount or goods below market value offered for the purpose of advertisement of tobacco and tobacco products. Ban on sales to minors. Ban on sale and storage within educational institutions and within 50 m of boundary of educational institutions.	Use is prohibited at places of public work or use, and in public transport vehicles. Ban on import of shisha (tobacco and non-tobacco) and related substances. Ban on print, electronic and outdoor media advertising of tobacco and tobacco products. Ban on free goods, cash rebates, free samples, discount or goods below market value offered for the purpose of advertisement of tobacco and tobacco products. Ban on sales to minors. Ban on sale and storage within educational institutions and within 50 m of boundary of educational institutions.
Turkey (EUR)	2004	No SLT products are licensed for sale. Single text warning required on 65% front/65% back. SLT products are included in definition of tobacco products in national tobacco control law. Impose excise duty (ad valorem and specific) and value added tax on chewing tobacco and snuff.	Combined pictorial/text warnings required on 65% (front) and 65% (back) of WP tobacco and front and back surface of WP water bowl/bottle. National tobacco control law includes WP (and non-tobacco WP) in definition of tobacco products. Use of WP banned in all indoor public places, workplaces and forms of public transport. Ban on sales to minors. Impose excise duty (ad valorem and specific) and value added tax on WP tobacco.
Iran (Islamic Republic of) (EMR)	2005	Ban on sale and import of SLT products.	Ban on use of WP in public places. Licence required for supply and sale.
UK (EUR)	2004	Ban on sale of oral tobacco (excluding chewing tobacco). Regulated under The Tobacco and Related Products Regulations 2016 in accordance with the EU Tobacco Products Directive. Single text health warning required on 30% front/30% back. Impose specific excise tax on chewing tobacco.	No WP-specific legislation; however, The Tobacco and Related Products Regulations 2016 cover all tobacco products. Requires combined pictorial/text warning on the front and back of the pack of WP tobacco. Impose specific excise tax on WP tobacco.
Bangladesh (SEAR)	2004	Pictorial health warnings required on 50% front/50% back. National tobacco law applies to all sucked or chewed tobacco products. Impose ad valorem excise and value added tax/sales tax on chewing tobacco. Ban on sales to minors.	National tobacco control law applies to tobacco products which can be inhaled through smoking. Pictorial health warnings required on 50% front/50% back of WP tobacco. Ban on sales to minors.
Sri Lanka (SEAR)	2003	Ban on manufacture, sale and import of SLT.	
Philippines (WPR)	2005	Pictorial health warnings required on 50% front/50% back. National tobacco law applies to tobacco products for the purposes of sniffing, sucking or chewing. Complete provisions on banning sales to minors, including all provisions under article 16.^1^ Impose specific excise on chewing tobacco.	Subnational policies in place. Ban on sales to minors.
Republic of Korea (WPR)	2005	Combined text/pictorial health warnings required on at least 50% front/50% back (Pictorial >30%). Ban on national TV and radio ads, international TV and radio, billboard and outdoor advertising, internet advertising. Health promotion fund and specific excise tax imposed on chew, snuff and snus imposed on SLT. Impose specific excise and value added tax/sales tax to snus. Ban on sales to minors.	Combined text/pictorial health warnings required on at least 50% of the package (Pictorial >30%). National smoke-free law applies to WP. Health promotion fund and specific excise tax imposed on WP tobacco. Ban on sales to minors.

1. Campaign for Tobacco-Free Kids. Tobacco Control Laws. 2018. https://www.tobaccocontrollaws.org/.

2. WHO report on the global tobacco epidemic, 2017. Monitoring tobacco use and prevention policies. Geneva: 2017.^2^

3. Mehrotra *et al* SLT control policies and their implementation. Noida: 2017.^12^

4. WHO FCTC Implementation Database. 2018. http://www.who.int/fctc/reporting/party_reports/en/.

5. Framework Convention on Tobacco Control FCTC-Secretariat- Implementation reports submitted by the parties http://untobaccocontrol.org/impldb/parties/.

FCTC, Framework Convention on Tobacco Control; TV, television.

## Regulatory experience of countries and WHO regions

### The Southeast Asia and Western Pacific regions

In Southeast Asia, the prevalence of SLT use is the highest in the world.^4 14–16^ Several countries of the region, such as India, Bangladesh, Sri Lanka and others, have taken strong measures to counter the growing use of these products.^17^ For example, under the Tobacco Control of Advertisements and Sale Act, Singapore has banned chewing tobacco since 1993 and in July 2010, an amendment expanded the scope of this act to encompass novel and emerging forms of tobacco products, such as tobacco derivatives (dissolvable tobacco) and nicotine-based products.^18^ Singapore has taken the lead in this region to implement FCTC articles 9 and 10 by establishing a laboratory for testing contents and emissions of cigarettes and measuring nicotine content in SLT products such as chewable tobacco, betel quid and khaini. In India, the health ministry has mandated the display of pictorial health warnings covering 85% of the principal display areas on all tobacco products as of 1 April 2016 and as of 2018, a new set of pictorial warnings is to be displayed on all tobacco products.^17 19^ Under the new rules, manufacturers will now need to display graphical pictures of throat cancer on cigarette and bidi packets and pictures of mouth cancer on chewing tobacco packets. In 2017, India has established three reference tobacco product testing laboratories.^11^ In Sri Lanka in 2015, the National Authority on Tobacco and Alcohol mandated rotating pictorial warnings that cover 80% of surface area (principal areas of both front and back of packs) of all tobacco products.^20^ It also prohibited the manufacture, import or sale of SLT tobacco products, e-cigarettes containing tobacco and cigarettes that are flavoured, coloured or sweetened.^20^ In the Western Pacific region, China does not have specific regulations that target NCTP. A permanent ban on SLT products (chewable, oral snuff, paste and/or powders) has been in effect in Australia since June 1991.^21^

## The European region

Regulations governing the use of SLT vary widely within Europe. In European Union (EU) member countries, SLT is regulated under EU Tobacco Products Directive 2001/37/EC, which prohibits the sale of tobacco for oral use. The EU Directive defines ‘tobacco for oral use’ as ‘all products for oral use, except those intended to be smoked or chewed, made wholly or partly of tobacco…particularly those presented in sachet portions or porous sachets, or in a form resembling a food product’.^22^ Sweden is an EU member but was exempted from regulations regarding the manufacturing, sale and marketing of snus within its borders. Furthermore and for manufacturing purposes, a voluntary quality standard for Swedish snus, named GothiaTek, has been used by the industry; it has set upper limits for selected toxicants in SLT.^23^ In Norway and because of the dramatic increase in snus use among young people in the past 10–15 years, cigarettes, roll-your-own and snus must be in plain packaging starting in 2017.^24^

In many Eastern European countries, SLT use is very low but still subjected to regulations regarding advertising and health warnings similar to those of smoked tobacco products. There has been an alarming increase in WP use in Latvia, the Czech Republic, Estonia and Slovenia.^5 25^ The Russian Federation has set a timetable to put into effect all FCTC articles by 2015, including a ban on the use of snus.^26^ Moreover, several Eastern European countries have joined the EU (or are in the process of joining) and consequently must observe the existing EU directive regarding SLT use.

In Turkey, where WP use has been historically engraved in its culture, laws against WP use have extended the definition of WP as follows: ‘all kinds of nargile and cigarette that do not contain tobacco but are used in a way to imitate the use of tobacco products shall be deemed as tobacco products’- Law no. 6487, 24/5/2013, article 26.^27^ Moreover, Turkey has mandated that warning labels should be placed on the bottles or bowls of the WP at public places and coffee shops.^28^ These measures have contributed to the observed drop in WP use in the country.^28^

## The Americas region

### USA

The Family Smoking Prevention and Tobacco Control Act, enacted in 2009, enables the US Food and Drug Administration (FDA) to regulate the manufacture, sale and distribution of tobacco products, including SLT and WP products.^29^ Provisions of the law include manufacturer registration and product listing requirements, warning labels, and enforcement of a minimum-age-of-sale restriction. The FDA has authority to set tobacco product standards including, for example, imposing limits on the amounts of nicotine, toxicants and/or additives that will be permitted in SLT products. In 2017, it proposed an N-nitrosonornicotine limit in finished SLT products.^30^ The FDA is also examining the public health impact of novel smokeless/dissolvable tobacco products and WP tobacco products.

### Canada

Generally, the prohibitions and requirements for tobacco products defined in Canada’s Federal Tobacco Act apply to SLT products, including the prohibition of selling tobacco to youth, restrictions on promotion and requirements for reporting by manufacturers.^31^

The labelling regulations, known as the 2000 Tobacco Products Information Regulations, also apply, but only to chewing tobacco, nasal snuff and oral snuff. For these classes of products, the regulations require text-based health warnings that occupy at least 50% of the principal display surfaces. Several Canadian jurisdictions have banned waterpipe smoking in public places. Recently, the Court in Ottawa upheld the city’s ban on smoking hookah pipes in public places which businesses considered a violation to the Charter of Rights and Freedoms.^32^

### Brazil

Despite the low consumption of SLT products in Brazil, regulatory authorities have detected a slight increase in the use of other tobacco products, including SLT and WP products, since the passage of a 2007 law, Regime Diferenciado de Contratações Públicas 090/07. Tobacco companies or importers must submit information about tobacco product contents and emissions, packaging, and design features. Brazil requires that SLT products be registered with the Brazilian health surveillance agency, Agência Nacional de Vigilância Sanitária, in order to be sold within the country, but as of 2012 no SLT products are registered, which means that they cannot be legally sold. By law, SLT products should carry warning labels and additives have been banned in the country.^33^

## The Eastern Mediterranean region

Interventions and regulatory policies regarding SLT and WP product use are not well structured in the Eastern Mediterranean region. Because of the historically high and further growing rate of use of WP in this region,^25^ countries have started in recent years to introduce regulations as recommended by FCTC articles and guidelines to combat this epidemic. The Emirate of Sharjah of United Arab Emirates (UAE) has a total ban on use of WP in public places and coffee shops. Several countries in the region, such as Islamic Republic of Iran, Egypt, Jordan, Syria and others, have incorporated WP use in their national tobacco control programmes.^34^ There is greater emphasis on banning use in public places and advertising, requiring textual and pictorial warnings, and education and awareness campaigns, but less so on raising taxes. Egypt has mandated pictorial warnings on WP tobacco packages, but it did not address the fact that many smokers are not exposed to these warning at time of use.^35^ Afghanistan has undertaken an aggressive campaign to combat WP use by increasing taxation, banning advertising and use in hotels and restaurants in Capital Kabul, Nangarhar and Herat provinces, and conducting awareness campaigns and posting warning signs in public places.^36^

In this region, SLT use is mostly observed in Pakistan, Afghanistan, Sudan, Yemen, and parts of Saudi Arabia and Egypt. Only Bahrain and the UAE have introduced policies banning SLT and SLT sales, partly targeting labour force in these countries. In 2008, Ajman Municipality in the UAE banned the sale, import, storage and possession of SLT and imposes heavy fines on violators.^4^ In 2009, the government of Bahrain introduced strong antismoking regulations and a law that prohibits the importation of SLT products.

## The African region

Despite increasing prevalence of SLT use, the countries of sub-Saharan Africa have limited SLT regulations and programmes.^4^ Tanzania has banned the use of SLT since 2006 and the Democratic Republic of Congo subjects SLT to a higher taxation scale than cigarettes.^4^ In South Africa, text warning labels on SLT cover 15% of the principal display area which is less than the 50% suggested by FCTC Article 11.^37^ A major challenge to tobacco control in the AFRO region is the prevalent use in many countries of SLT products produced by a cottage industry which distributes and markets them on a local rather than national or international scale. Collecting relevant data and information about importation and use of SLT in African countries is important to helping these countries develop their capacity to regulate SLT products. WP use has been on the rise in this region, particularly among youth; in recent years, Kenya, Tanzania and Rwanda have banned the use of WP.^4 38 39^

## Discussion

The above descriptions on country, regional and global experiences indicate the increased awareness about the importance of incorporating NCTP in national tobacco control measures as the FCTC treaty stipulates. Indeed some progress is observed and documented but it stops short of a comprehensive implementation of the FCTC articles to their regulatory endpoint. The published literature assessing the implementation of FCTC articles to control NCTP use and the impact of these measures corroborates the above-described global and regional reports with the strongest achievements observed in protection from exposure to tobacco smoke (article 8); advertising, promotion and sponsorship (article 13); sales to and by minors (article 16); education, communication and public awareness (article 12) and packaging and labelling of tobacco products (article 11).^12^ Such conclusion is affirmed by Cornacchione Ross *et al* who conducted a systematic review of 45 studies on health communication for NCTP^40^; these capitalised on health warnings and communication campaigns for SLT (71.1%), WP tobacco (20%), electronic nicotine delivery systems (4.4%), cigars (4.4%) and potentially reduced exposure products (2.2%). These studies most commonly examined tobacco product warnings (57.8%), public education (42.2%), which included mass media campaigns, knowledge, attitudes and beliefs as outcomes (60%), but behaviour was an outcome in the minority of studies (17.8%).

To identify gaps in implementing key SLT demand-reduction measures, Siddiqi *et al* confirmed the limited content of the published literature and pointed out the fact that most studies were conducted outside Southeast Asia, the geographical region with the highest SLT prevalence of use.^41^ They concluded that the literature supports that some SLT demand-reduction measures have been implemented; however, for taxation, labelling and packaging, most administrations have weaker policies for SLT than for cigarettes. They highlighted the gap in regulating SLT contents and the lack of supportive cessation programmes.

In addressing the implementation of articles 9 and 10 relevant to NCTPs, the lack of sufficient information on contents and emissions of the heterogeneous SLT and WP tobacco products has been of concern and interest to the COP and the Study TobReg. Over the past few years, the latter scientific group has published several technical reports on SLT that can serve as basis for the implementation of FCTC articles 9 and 10. TobReg recommendations called for setting limits for carcinogens in SLT, reducing the use of *Nicotiana rustica*, air curing rather than flue curing of tobacco during manufacturing, pasteurisation of tobacco as compared with fermentation and storage under controlled humidity and temperature to prevent microbial overgrowth.^42^ Moreover, the Tobacco Laboratory Network^43 44^ is currently validating the application of its published standardised operating procedures for measuring selected contents and emissions of cigarette tobacco to WP tobacco and SLT products. As for WP, TobReg emphasised that emissions depend on the tobacco product smoked, and on the combination of tobacco product, charcoal type, WP design, WP preparation method, puff topography and their interactions.^44^ Therefore, public health protection requires regulation of the characteristics and contents of WP tobacco products and charcoal.^5^ Many countries are offering cessation programmes for tobacco use as stipulated in FCTC article 14, but experience with NCTP remains limited; only few studies have been published for this purpose with limited evidence of effective outcome.^45–52^ A review by Maziak *et al* of three WP cessation intervention trials concluded that these studies set the way for developing future interventions that build on experience of cigarette cessation trials and take into consideration the social dimension and unique components of WP smoking and assessment tools.^45^ Similarly, the experience in cessation programmes for SLT tobacco shows the high rate of quit attempts with a low success rate. The published studies highlight the importance of addressing cultural practices, awareness of health effects, social support, and reinforcing legislation and control for successful cessation programmes.

As described above, the progress in applying FCTC to NCTP is documented in global and country reports and the literature^28 53–60^; however, serious limitations are observed and these can be attributed to the following factors:

1. Unique nature and design features of these products. For example, the pictorial or text warning labels on WP tobacco product is not visualised by the smoker at the time of smoking the WP.^61^ Many SLT products, such as paan, gutka and zarda in Pakistan, India and Bangladesh or toombak in Sudan or shammah in Yemen, are sold on a non-packaged individualised basis with no room for warning labels.^4^
2. Deeply rooted cultural practices typically associated with cottage industries that do not adhere to standardised manufacturing practices rendering it difficult to regulate. This is especially true for SLT, bidis and WP tobacco.^4 62–64^
3. Insufficient information on the contents and emissions of many of these products and lack of conclusive research evidence on effective control measures and regulations against their use.^4 65^
4. The exclusion of NCTP from the increased taxation schemes that were progressively applied over time to the sale of cigarettes.^66 67^ For example, the literature affirms low tax rates and commensurate affordability of NCTP in low-income groups.^64 68 69^
5. Weak enforcement of tobacco control measures.^17 70 71^ For example, the failure to prohibit the use of flavourings in manufacturing these products which makes them very attractive to youth and thus promotes use.^72 73^
6. The adoption of generic definitions for tobacco products and smoking that do not specify NCTP. In the case of WP, such ambiguity has allowed the tobacco industry to take advantage of the situation by promoting misleading information about the health effects of WP use,^66 74^ operating fashionable and trendy establishments such as hookah or shisha bars,^75^ using internet and social media to promote its products,^76 77^ and undermining tobacco control policies for NCTP sales and promotion in the globalised market.
7. The inadequacy of cessation interventions to address the deeply rooted cultural acceptance of NCTP across all ages and genders.^4 45 50 72 73 78^

The FCTC has been a catalyst for action and effective control of all tobacco products, including NCTP, but more action is needed against the latter. In recent years, there has been greater attention and emphasis on such products in national tobacco control plans with legislative and administrative measures that implemented most of the key demand reduction articles of the FCTC. Regulatory measures, such as plain packaging and standardised packaging of NCTP and placing warning labels on WP parts and accessories, can potentiate the effectiveness of these demand reduction measures. However, several challenges remain, most importantly the deeply rooted cultural practices which prevail in low-income and middle-income countries where unfortunately reinforcing regulations are frequently lax and increasing taxation as one of the effective control measures has not been practised to the extent applied to cigarettes. Legislation remains insufficient in addressing cross-country trade, internet purchases and social media promotions, and health risks particularly to adolescents and young adults who are most at risk. Cessation programmes on NCTP are in their early stages and more research is needed to make effective recommendations that take into consideration the peculiarities of the products and the social norms associated with their use.

Because of the challenges in regulating NCTP and applying FCTC articles to their use, the Convention Secretariat of FCTC facilitated the building of global networks to enhance policy action against all tobacco products, including NCTP, and to create forums for training, sharing best practices and successes regionally and internationally.^79^ These efforts encompassed the establishment of seven global knowledge hubs and in 2017 with two specifically targeting NCTP; the first on SLT at the National Institute of Cancer Prevention and Research in Delhi, India^11^ and the second on WP at the American University of Beirut in Lebanon.^34^

In brief, FCTC provides an instrument to control all tobacco products including NCTP. Thus far, its implementation to control NCTP is limited to some of the demand reduction articles and there should be a concerted global effort by parties to incorporate explicitly NCTP in their national tobacco control plans and to expand the scope of regulations to apply more of FCTC articles to their use and demonstrate commitment to the reinforcement of such measures. With the absence of a comprehensive legal database on NCTP regulations as well adequate assessment and evaluation of already implemented policies, it would be fruitful to establish and maintain such database to be shared by parties facing the growing challenge of regulating NCTP and for researchers to investigate which measures are most effective in controlling their use.

**What this paper adds**

- It is an update of the achievements made thus far by parties in applying Framework Convention on Tobacco Control (FCTC) articles to non-cigarette tobacco product (NCTP) use, and highlights the need of their policy-makers to reinforce stronger regulations, increase taxation and address the wide social and cultural acceptance of NCTP, specifically among youth and adolescents.
- This is a narrative assessment of the impact of WHO-FCTC in controlling NCTPs since its adoption in 2005.
- It reports on 12 country visits and reviews 412 publications. Global progress has been achieved primarily in implementing demand reduction FCTC articles.
- It highlights the challenges in applying all FCTC articles to NCTP use and the gaps to be targeted by regulators and policy-makers.
